# Comparative Review of Brucellosis in Small Domestic Ruminants

**DOI:** 10.3389/fvets.2022.887671

**Published:** 2022-05-12

**Authors:** Carlos Alberto Rossetti, Estefanía Maurizio, Ursula Amaranta Rossi

**Affiliations:** ^1^Instituto de Patobiología Veterinaria, Instituto Nacional de Tecnología Agropecuaria (INTA)-Consejo Nacional de Investigaciones Científicas y Técnicas (CONICET), N. Repetto y de Los Reseros, Buenos Aires, Argentina; ^2^Consejo Nacional de Investigaciones Científicas y Técnicas (Conicet), Buenos Aires, Argentina

**Keywords:** *Brucella melitensis*, *Brucella ovis*, genomics, goats, sheep, pathogenesis

## Abstract

*Brucella melitensis* and *Brucella ovis* are the primary etiological agents of brucellosis in small domestic ruminants. *B. melitensis* was first isolated in 1887 by David Bruce in Malta Island from spleens of four soldiers, while *B. ovis* was originally isolated in Australia and New Zealand in early 1950's from ovine abortion and rams epididymitis. Today, both agents are distributed worldwide: *B. melitensis* remains endemic and associated with an extensive negative impact on the productivity of flocks in -some regions, and *B. ovis* is still present in most sheep-raising regions in the world. Despite being species of the same bacterial genus, *B. melitensis* and *B. ovis* have extensive differences in their cultural and biochemical characteristics (smooth vs. rough colonial phases, serum and CO_2_ dependence for *in vitro* growth, carbohydrate metabolism), host preference (female goat and sheep vs. rams), the outcome of infection (abortion vs. epididymitis), and their zoonotic potential. Some of these differences can be explained at the bacterial genomic level, but the role of the host genome in promoting or preventing interaction with pathogens is largely unknown. Diagnostic techniques and measures to prevent and control brucellosis in small ruminants vary, with *B. melitensis* having more available tools for detection and prevention than *B. ovis*. This review summarizes and analyzes current available information on: (1) the similarities and differences between these two etiological agents of brucellosis in small ruminants, (2) the outcomes after their interaction with different preferred hosts and current diagnostic methodologies, (3) the prevention and control measures, and (4) alerting animal producers about the disease and raise awareness in the research community for future innovative activities.

## Introduction

Brucellosis is a worldwide, chronic infectious disease caused by small aerobic, non-motile, Gram-negative coccobacilli of the genus *Brucella*. There are 12 established species within the genus that are recognized based on preferential host specificity (1). Goats and ewes are the preferred hosts for *Brucella melitensis* whereas rams are for *Brucella ovis*, although small domestic ruminants may be infected by other *Brucella* species.

*B. melitensis* infection causes abortion, stillbirths and the birth of weak offspring, and occasionally epididymo-orchitis in goats and sheep (2) and is the most virulent *Brucella* species for humans (zoonotic), responsible for a severely debilitating and disabling illness that results in high morbidity with low mortality (3).*B. melitensis* has been controlled in most industrialized countries; however, it remains endemic and associated with an extensive negative impact on the productivity of flocks in low and middle-income nations, where goats and sheep are the major livestock species and the main economical livelihood, such as the Mediterranean region, the Middle East, Central Asia, Sub-Saharan Africa, and parts of Latin America (4).

**Figure 1:**
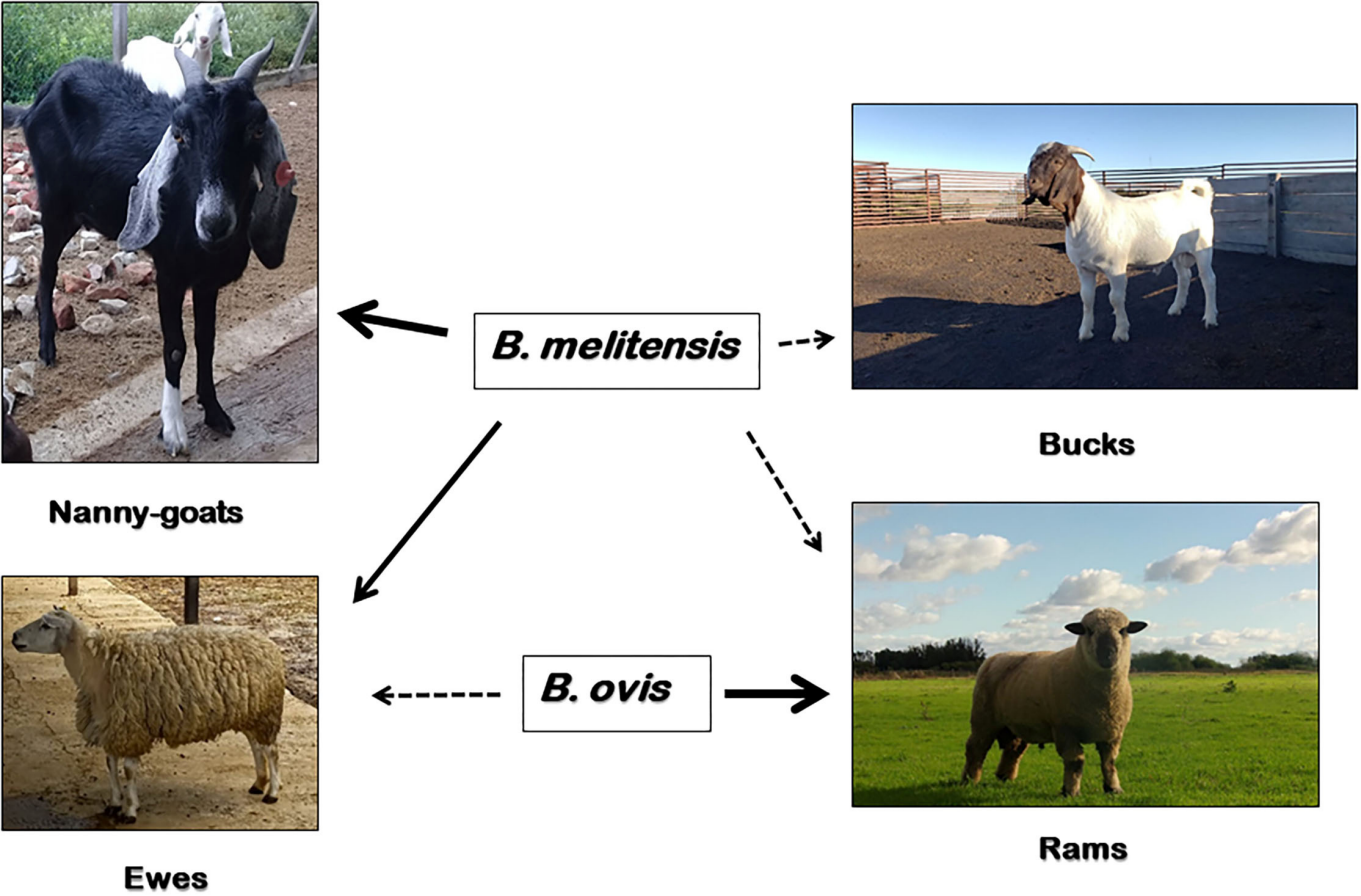
Susceptibility of domestic small ruminants to *B. melitensis* and *B. ovis* infection. *B. melitensis* cause abortion, stillbirths and the birth of weak offspring in goats and sheep (solid arrow); and less commonly, epididymo-orchitis in bucks and rams (dashed arrow). Moreover, *B. ovis* affect rams almost exclusively (solid arrow), causing epididymitis and occasionally abortions in ewes and an increase in perinatal death (dashed arrow). In addition to sheep and goats, *B. melitensis* has a wide range of natural susceptible host species, including humans. Other than domestic sheep, *B. ovis* has only been isolated from naturally-acquired infections of farmed red deer (*Cervus elaphus*), but experimental infections have been established in goats, Rocky Mountain bighorn sheep (*Ovis canadensis canadensis*) and white-tailed deer (*Odocoileus virginianus)*.

On the contrary, *B. ovis* seems to be non-pathogenic for humans and the main clinical sign of infection is epididymitis in rams, with occasional abortions in ewes and increased perinatal death (5) (Figure 1). In small ruminants, there are reports of *B. ovis*-specific antibodies in goats from Brazil (6) and Bulgaria (7), and evidence of seroprevalence in Rocky Mountain bighorn sheep (*Ovis canadensis canadensis*) in the USA (8). However, the etiological agent has only been isolated in naturally-infected domestic sheep and farmed red deer (*Cervus elaphus*) (9). Experimental infection of male goats with *B. ovis via* a natural route of entry showed that bucks may become infected and shed the pathogen in semen, but the infection is transient and their role in the epidemiology of the disease is negligible (10, 11). *B. ovis* was first isolated and identified from ovine abortion and epididymitis in rams in Australia and New Zealand in early 1950's (12, 13). Following these first reports, *B. ovis* has been found worldwide distributed. Today, the disease probably occurs in most sheep-raising regions in the world, being currently present in, e.g., Australia, New Zealand, Russia, France, Spain, Portugal, South Africa, United States, Mexico, Argentina and Brazil (14).

In addition, small domestic ruminants may also be infected by other *Brucella* species. Goats and sheep may be susceptible to *B. abortus* infection under particular epidemiological situations (for instance, when they live in close contact with *B. abortus*-infected cattle or camelids) (15–17); however, these flocks will not sustain the infection in the absence of infected primary host (18). *B. suis* isolates from goats and sheep have been seldom reported (19–22), and to the best of our knowledge, no other *Brucella* species have been isolated from small domestic ruminants. Considering that small ruminants may act as occasional hosts for other species of the genus *Brucella*, this review will focus on brucellosis caused by *B. melitensis* and *B. ovis*. Throughout this review, we will present: (1) the differences and similarities of the etiological agents at the biochemical and genomic level, (2) the pathogenesis and clinical consequences in their primary hosts, (3) the indicative prevention and control measures for the disease, and (4) summary and analysis of current guidelines and awareness tools to alert animal producers about the disease and promote future innovative actions in the research community.

## Cultural and Biochemical Differences Between *B. melitensis* and *B. ovis*

Bacterial isolation remains the gold standard for diagnosis of brucellosis. Isolation of *Brucella* is typically achieved from fresh milk samples and vaginal discharges of sheep and goats (2), while samples from lymph nodes (LNs), spleen, reproductive tract and udder are collected for culture at necropsy (23). On the other hand, semen is the optimal sample for *Brucella* isolation in rams and goat bucks, whereas inguinal LNs, spleen, seminal vesicles and the epididymis are the preferred necropsy samples (24). The abomasal content and lungs, followed by the liver and spleen, are the preferred samples for *B. melitensis* and *B. ovis* isolation from an aborted fetus (2).

*B. melitensis* grows in non-enriched basal media such as *Brucella* medium base, tryptose -or tripticase- soy agar (TSA), blood agar base or Columbia agar, without serum or CO_2_ enrichment (25). Contrarily, optimal growth of *B. ovis* occurs when culture media is enriched with 5–10% blood or serum (sheep > bovine or horse > fetal calf serum) and incubated at 37°C in a microaerophilic atmosphere (i.e., 10–20% CO_2_). This CO_2_-dependence of *B. ovis* is caused by a guanine insertion in BOV_RS08635 locus that expresses a carbonic anhydrase II defective enzyme (26). Unlike *B. melitensis, B. ovis* cannot grow on glucose or galactose as primary carbon source, and is defective in metabolisms of ribose and erythritol (27). Selective media containing antibiotics are effective in suppressing contamination, and therefore useful for the isolation of field specimens. In spite of being the most widely used selective media for primary *Brucella* isolation, Farrell's medium contains antibiotics in its composition that may inhibit the development of *B. ovis* and some *B. melitensis* strains (28). On the contrary, other selective media such as modified Thayer-Martin (mTM) or Skirrow agar facilitate *B. ovis* and *B. melitensis* isolation, although the control of overgrow of contaminating microorganisms is less stringent (29). A more sensitive selective medium (CITA medium) for primary *Brucella* isolation from field veterinary samples, was more recently developed (30). This new culture medium, in comparison to mTM has a 7-fold higher concentration (mg/L) of a Gram-positive bactericidal antibiotic vancomycin, and the addition of antifungal agent amphotericin B. A presumptive fast bacteriological diagnosis can be made on smears from placentas, aborted fetuses or vaginal swabs stained with Stamp's modification of the Ziehl-Neelsen's technique, although this method is not specific and the results must be confirmed by culture or PCR (25).

On solid media, 3–4-day old *B. melitensis* colonies are convex, translucent, smooth, glistening, and circular, 0.5 to 1 mm in diameter, while *B. ovis* colonies are round, more opaque with a dry, yellowish-white granular (rough) appearance and up to 2 mm in diameter (25). This difference is because *B. melitensis* expresses a full lipopolysaccharide molecule (smooth, S-LPS) that is anchored in the outer membrane (OM), while *B. ovis* expresses LPS that lacks the O-antigen (rough, R-LPS). However, bacterial culture identification has a low sensitivity, is time consuming, and requires skilled technical personnel to safely handle samples and live bacteria. Moreover, *B. melitensis* is associated with biosafety concerns due to the high risk of acquired laboratory infection and must be handled under BSL3 conditions, making diagnosis by bacterial isolation impractical in many situations. As opposed to *B. ovis* which does not require biosafety measures due to its lack of zoonotic potential, isolation and handling can be conducted at BSL2 facilities.

*B. melitensis and B. ovis* are identical at the microscopic level: both are small coco-bacillus with a size of 0.5–0.7 um in width and 0.6–1.5 um in length, non-capsulate, non-motile, and non-spore-forming, single or rarely forming short chains (25). For routine identification, only a few biochemical tests such as oxidase, urease, growth in presence of colorants and agglutination with monospecific serum are indicated to differentiate between both species. The main differences between these two *Brucella* species are summarized in Table 1.

**Table 1:** Main differences between *B. melitensis* and *B. ovis*.

**Characteristics**		***B. melitensis***	***B. ovis***
Host preference		Goats and sheep	Sheep (Ram)
Host susceptibility		Goats, sheep, cattle, human	Sheep, red deer
Zoonotic capacity		High	None
Biosecurity requirements		BSL 3	BSL 2
Biovars		3 (1–3)	1
Colony morphology		Smooth	Rough
CO_2_ dependence		–	+
Serum/blood requirement		–	+
Oxidase		+	–
Urease activity		+ (most strains)	–
Growth on	Basic fuchsin	+	Growth inhibited at ≥20 ug/ml (1/20.000)
	Methyl violet	+	–
Agglutination	Monospecific serum	Biovar 1: M	R (anti-rough)
		Biovar 2: A	
		Biovar 3: A–M	
	Acriflavine (1:1,000)	–	+

## Pathogenesis of Brucellosis in Small Ruminants

Susceptibility to *B. melitensis* infection increases from naïve sexually immature to sexually mature animals of either sex, and reaches its maximum in pregnant goat does and ewes. It is well-known that goats are more susceptible than sheep, and rams more resistant than ewes to the development of brucellosis disease caused by *B. melitensis*. A great variability in susceptibility to brucellosis was reported between sheep breeds, but not between breeds of goats. For example, Hampshire down and Texel breeds are more resistant to infection and consequently less likely to abort, than some dairy breeds and Southwest Asia and Mediterranean fat-tailed breeds (3, 31).

**Figure 2:**
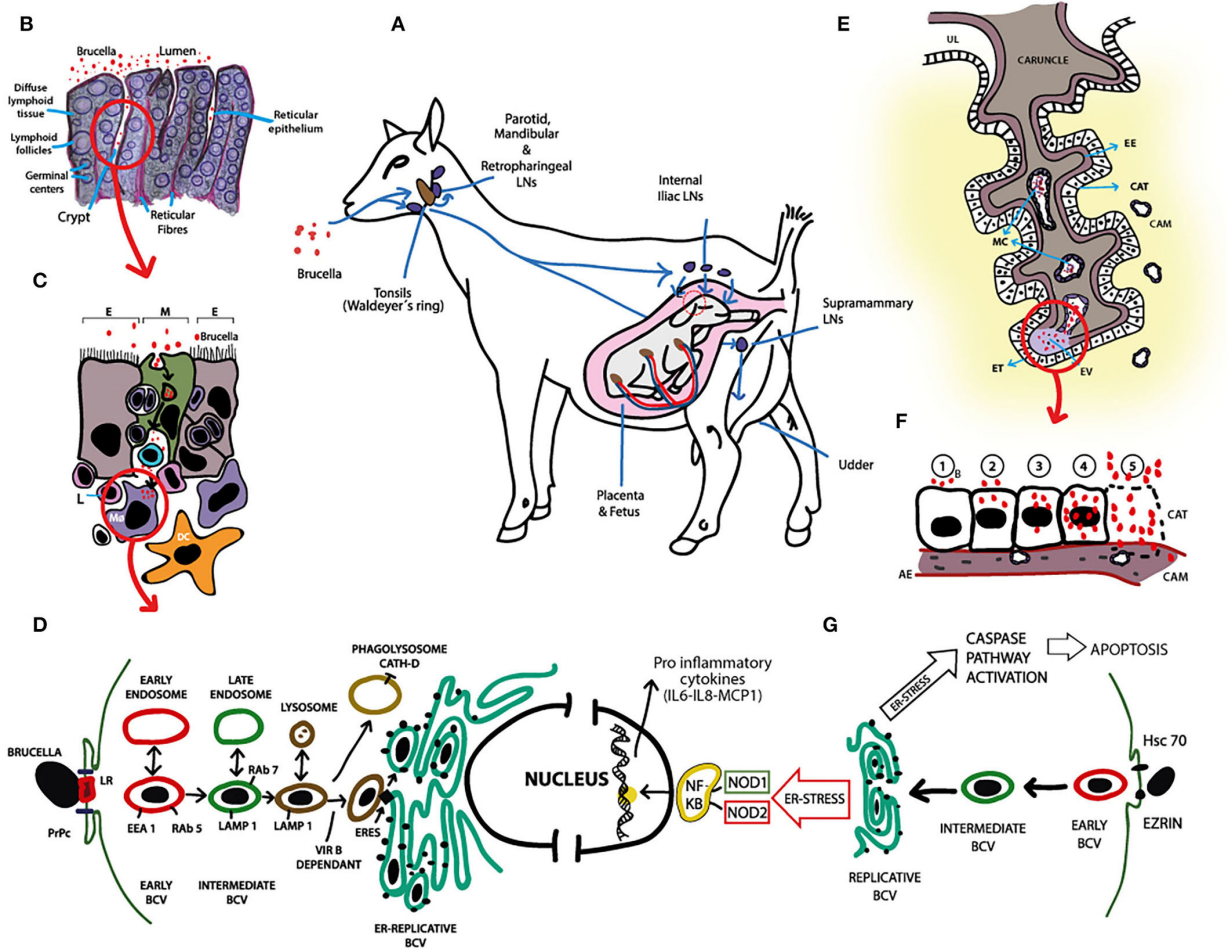
Pathogenesis of *B. melitensis* infection in small ruminants. **(A)** *B. melitensis* infects small ruminants mainly *via* the alimentary tract. Once in the oral cavity, *B. melitensis* enters through the mucosa or the oro- and nasopharyngeal tonsils (Waldeyer's ring) and colonizes, proliferates and persists in the lymph nodes (LNs) of the head (i.e., mandibular, parotid, and lateral and medial retropharyngeal LNs). Failure to eliminate *B. melitensis* on this primary line of defense results in escape of the bacteria through the efferent lymphatic vessels to the distal LN or *via* blood to the general circulation. In pregnant females, *B. melitensis* colonizes placenta and induces abortion or stillbirth. *Brucella melitensis* also has affinity for lactating udder. Significant numbers of *B. melitensis* are excreted in vaginal discharges, aborted fetus, placenta and milk. **(B)** Illustration of histological structure of tonsils. *B. melitensis* is first seen in the lumen of the oral cavity. Subsequently, *B. melitensis* colonizes the crypts and invade through reticular epithelium. **(C)** Reticular epithelium is composed of scattered M cells (M; green), lymphoid cells and the epithelial cell types (E). M cells endocytose *B. melitensis* from the lumen, after which transcytosis and basolateral release occurs. Immediately, the agent is up taken by resident antigen presenting cells, i.e., macrophages (MØ) and dendritic cells (DC), which in turn activate the immune response in the underlying lymphoid follicles. MØs and DCs traffic *B. melitensis* to other sites in the body or back through the reticular epithelium to the mucosal surface. **(D)** Schematic representation of *B. melitensis* intracellular trafficking in macrophages and evasion of killing. Following lipid rafts (LR)—and the prion protein (PrPc)—mediated internalization, *B. melitensis* is contained in an early vacuole (BCV: *Brucella* containing vacuole). This early vacuole sequentially interacts with cellular organelles (early and late endosomes, and lysosomes) and transiently acquires different markers (EEA1, Rab5, LAMP1, Rab7) in a *VirB* dependent-mechanism regulated by the pathogen effector proteins. To reach the replicative niche, BCV-LAMP1+ interacts with ER exit sites (ERES), and generates an ER-derived organelle permissive for *B. melitensis* replication (ER-Replicative BCV). Vacuoles containing *VirB*-deficient *B. melitensis* undergo phagolysosomal degradation. Spontaneous rough mutant generation might help parental pathogen release from infected MØ through lytic or non-lytic mechanisms, and the process repeats in other professional phagocytic cells. **(E)** Diagram of ruminant placentome with enlargement of one caruncular septa. *Brucella* invades placenta *via* maternal capillaries (MC). Extravasated (EV) maternal blood at the tip of the caruncular septa, along with *B. melitensis*, is phagocytosed by erythrophagocytic trophoblasts (ET). From these cells, *B. melitensis* spread infection to adjacent chorioallantoic trophoblasts (CAT). UL: Uterine lumen, EE: Endometrial epithelium, CAM: Chorioallantoic membrane. **(F)** Schematization of a complete cycle of *B. melitensis*-infected chorioallantoic trophoblast (CAT). 1: *B. melitensis* (B, red circles) adheres to the plasmatic membrane of CAT; 2: the bacteria are internalized and initial intracellular replication occurs; 3–4: Massive intracellular multiplication of the agent; 5: Apoptosis of CAT and release of huge number of *B. melitensis*. The cycle of endocytosis, intracellular replication and programmed cell death continues. CAM, Chorioallantoic membrane; AE, Allantoic ephitelium. **(G)** Schematic representation of *B. melitensis* intracellular trafficking in chorioallantioic trophoblasts. Trophoblasts invasion of *B. melitensis* is mediated by heat shock cognate protein 70 (Hsc70) and Ezrin. From this point until reaching of the replicative niche, intracellular trafficking is similar than that reported in macrophages (i.e., BCV goes from early BCV -red- to intermediate BCV -green- and to replicative BCV). Intracellular presence of *Brucella* induces ER stress that triggers production of the pro-inflammatory cytokines in a nucleotide-binding oligomerization domain (NOD) 1/2—dependent manner *via* activation of NF-kb pathway, and activates caspase pathway leading to chorioallantoic trophoblast apoptosis. These molecular responses of *B. melitensis*-infected trophoblasts may contribute to better understanding the pathogenesis of placentitis and abortion in small ruminant brucellosis.

The predominant route for *B. melitensis* infection under natural exposure is the alimentary tract (2). Animals, mainly goat does because of their naturally more curious character, are primarily infected by direct contact with aborted fetuses, placental membranes, vaginal discharges, or ingestion of contaminated water, pastures or colostrum. Once in the oral cavity, *B. melitensis* enters through the mucosal-associated lymphoid tissue (MALT) in the pharyngeal wall and colonizes, proliferates and persists for long periods of time in the lymph nodes (LNs) of the head (i.e., mandibular, parotid, and lateral and medial retropharyngeal LNs) (32). This MALT forms a ring around the pharyngeal wall called “Waldeyer's ring,” that presents a similar histologic structure than the ileal Peyer's patches, an anatomic structure easily penetrated by *B. melitensis* (33) (Figures 2A–C). Failure to eliminate *B. melitensis* at the primary line of defense results in bacterial escape through the efferent lymphatic vessels to the distal LNs or *via* blood to the systemic circulation (Figure 2A). *Brucella* spreads free in plasma, inside erythrocytes or within phagocytic cells and can reach every organ, but persists in lymphoid tissues (LNs and spleen) and bone marrow (Figure 2D) and actively replicates in the pregnant uterus or, less frequently, in testis and epididymis. As strategy for persistence, *B. melitensis* prevents the apoptosis of infected mononuclear phagocytes to facilitate bacterial persistence in the reticuloendothelial system until the host becomes susceptible to placental colonization and replication during pregnancy (34). Considering that *Brucella* can reach 1 × 10^10^ colony-forming units (CFU)/ml in allantoic fluid and 1 × 10^13^ CFU/g of tissue in cotyledons (35, 36), and the infective dose of *B. melitensis* is 10 to 100 CFU (37), abortion and excretion of *B. melitensis* in vaginal discharges constitute an important new source of infection for other susceptible hosts.

Contrary to *B. melitensis, B. ovis* primarily infects males, and almost exclusively rams after puberty (5). *B. ovis* infection seems to be transient in bucks (10, 11), rarely cause abortions in ewes (38), and has apparently no clinical consequences in goat does. Similar to the *B. melitensis* infection, susceptibility to *B. ovis* infection may vary among sheep breed. In South Africa, the prevalence of clinical lesions was rated as Dorper > Karakul > Merino, and other authors also support that Merino breed is more resistant than other breeds of sheep (39–41), although potential flock confounding variables were not fully controlled.

Flocks become infected by the introduction of a *B. ovis*-infected ram, which disseminates the pathogen intermittently in semen, genital secretions and urine. Indirect venereal transmission between rams that have mated with the same ewes, or direct non-venereal transmission by homosexual behavior, or by the sniffing or licking of the preputial area of the infected ram are the most important ways of spreading the infection (5, 42). The pathogenesis of infection in rams has been poorly studied, but it appears that after entering the body by penetrating the mucosae of the prepuce, rectum, oral or nasal cavity, *B. ovis* is phagocytosed by local antigen presenting cells, and transported to the regional lymph nodes. The possibility of infection *via* digestive route should not be considered, as lack of urease in *B. ovis* specie reduces its ability to survive in the acidic abomasal content (43). Progression of the disease is slow, and after 2–8 weeks of incubation period, the pathogen disseminates *via* lymphatic or blood vessels, free or in leukocytes, but in the long term only colonizes the genital organs and accessory sexual glands (seminal vesicles, bulbourethral—Cowper's—glands, ampullas of the *vas deferens*) (44). Colonization starts in the tail of epididymis and seminal vesicles, yet the molecular basis for targeting these tissues is unknown.

The pathogenesis of *B. ovis* infection in ewes has not been fully elucidated. Even though mating by infected rams or artificial insemination with infected sperm causes a low percentage of infection in ewes, contaminated semen may be the main source of infection of female sheep, as environmental contamination (water, pastures) does not seem to play a relevant role for *B. ovis* transmission (38, 45, 46), probably due to its deficient ability to infect *via* the oral route. After insemination with contaminated semen, *B. ovis* is transported and remain confined to the local drainage lymph nodes; later in pregnancy, bacteremia develops and *B. ovis* reaches the pregnant uterus and colonizes the fetus through chorion vessels. Opposite to what occurs with *B. melitensis* where subsequent reinvasion of the pregnant uterus and shedding may happen during following parturitions (2), *B. ovis* infection rarely extends from one pregnancy to the next (45, 47, 48). Therefore, the role of infected ewes in shedding and dissemination of the *B. ovis* is insignificant, as the ability of the pathogen to remain in the uterus or being excreted in vaginal discharges and milk seems to be limited, although exceptions were reported (49).

## Clinical Consequences of *BRUCELLA*-Infection in Domestic Goats and Sheep

### Abortion and Weak Offspring

Middle to late-term gestation abortion, stillbirths and the delivery of weak offspring sometimes followed by the retention of fetal membranes are the characteristic—and perhaps the only noticeable—clinical signs of the disease. However, these signs are not pathognomonic of brucellosis, and a differential diagnosis with other etiological agents is critical, although sometimes difficult to achieve in the clinical field. Therefore, it would be judicious to assume that all abortions are caused by a zoonotic agents and minimize exposure until a definitive diagnosis is made.

During bacteremia*, Brucella* enters the placenta through specialized trophoblastic cells located toward the fetal side, between the bases of cotyledonary villi of the placentomes that are involved in phagocytosis of macromolecules, especially extravasated maternal blood (Figure 2E). Thus, *Brucella* takes advantage of an important physiological mechanism for the trans-placental transport of iron needed by the developing fetus for erythropoiesis (50) to colonize naive targets. From these phagocytic trophoblasts, *Brucella* spreads and replicate into adjacent chorioallantoic trophoblasts (51). Massive intracellular multiplication induces apoptosis of trophoblasts due to endoplasmic reticulum stress (52) and release of huge numbers of microorganisms into the uterine lumen. Though, the cycles of endocytosis (or active penetration), intracellular replication and cell death continue (Figure 2F); consequently, placenta cotyledons heavily fill with brucellae and fetal invasion *via* bacteremia occur. Recent molecular studies demonstrated that *Brucella*-infected trophoblasts secrete proinflammatory chemokines such as IL6, IL8, GCP-2 and MCP-1, and hormones prolactin and estrogen, while the secretion of progesterone is inhibited (52–55) (Figure 2G). Altogether, these elements provide a local environment that contributes to abortion. Foci of apoptotic trophoblasts result in ulceration of the chorioallantoic membrane, which favors the hematogenous dissemination of *B. melitensis* to chorionic villi and fetal tissues (56, 57). Large numbers of brucellae in chorionic connective tissue gradually produce vasculitis and separation of fetal trophoblasts from maternal syncytial epithelium. Other histologic changes are associated with neutrophilic and histiocytic inflammatory infiltrate, and fibrin deposition, ranging from subacute to massive chronic purulent necrotizing cotyledonary placentitis. Eventually, when the number of viable placentomes is inadequate to sustain maternal-fetal interaction, fetal death and consequent abortion occurs. Fewer affected placentomes often results in underweight—weak newborns followed by a high neonatal mortality rate.

The mechanism that makes the pregnant uterus attractive to brucellae, especially to *B. melitensis*, is still undefined. High levels of erythritol in the ruminant placenta were originally postulated to explain the tropism and the subsequent accumulation of *Brucella* in this organ. In favor of this, the promoting role of erythritol on *B. melitensis* expression on virulence genes, such as the Type IV secretion system VirB and flagellar proteins was also more recently demonstrated (58). However, there is increasing evidence that *B. melitensis* infect placenta and produce abortion in hosts such as humans (59, 60), mice and guinea pigs (61, 62), where erythritol is not a major component of the placenta. A clue to this mystery might have been recently deciphered by Barbier et al., who assigned a crucial role to the host polyol pathway enzyme aldose reductase, which catalyzes the synthesis of several alternative carbon (C) sources (including erythritol) for *Brucella* availability (63). Contrarily, *B. ovis* is unable to catabolize erythritol and many other gluconeogenic substrates and use them as C sources (27, 43), which may explain why the pregnant uterus is not as attractive to this *Brucella* specie as it is for others, resulting in lower incidence of abortion in infected ewes.

### Epididymitis

*B. ovis* affect the epididymis, tunica vaginalis and testes producing infertility in rams. Earlier events include quantitative and qualitative alterations of semen characteristics (sperm motility, concentration, morphology, reduced fertility) which progress to palpable lesions in the scrotum with no changes in libido. Palpable lesions of the epididymis are occasionally bilateral and may vary from a slight enlargement to large indurations, with the tail affected more often than the head or the body (5). The testes may suffer atrophy and the tunica vaginalis is often thickened and fibrous, with extensive adhesions. Yet, *B. ovis*-infected rams may not always develop clinical manifestations; indeed, subclinical disease where seminal vesicles is the most common affected organ, appears to be the rule (42, 64). According to previous studies, *B. ovis* reach the genital tract by haematogenous spread, extravasate and colonize the interstitium. Earliest histologic changes are seen in the tail of epididymis, consisting in perivascular edema and diffuse interstitial accumulation of lymphocytes and plasmatic cells (65). Further degeneration and lysis of the epithelium allow extravasation and interstitial accumulation of spermatozoa, which increases the inflammatory response with tubular occlusion, formation of sperm granulomas and chronic pyogranulomatous epididymitis (64). From the time that *B. ovis* reaches the genital organs, the microorganism is chronically shed in semen and genital secretions, free or in infected phagocytes.

More rarely, *B. melitensis-*infected bucks and rams might present unilateral or bilateral epididymo-orchitis and can shed *Brucella* in semen for a year (66–68). Even though the epidemiological importance under natural conditions of the venereal route is usually disregarded, *B. melitensis*-contaminated semen used in artificial insemination could be a potential source of infection (69).

### Other Clinical Consequences

*B. melitensis*, as well as *B. ovis*, have marked affinity for the lactating udder and supramammary LNs, although clinical findings are limited to decreased milk yield and slight enlargement of the LNs (47, 70). *Brucella-* infected phagocytic leukocytes migrate from the systemic circulation to the mammary alveoli. In the ductal and alveolar lumen, *Brucella* replicates within phagocytes and upon host cells degeneration, brucellae are subsequently released and ingested by a new round of phagocytes. Part of the infected phagocytes is excreted in milk, and part migrates to the interstitium and reaches the supramammary LNs *via* the lymphatic stream (71). Milk components, like fat and casein, may reduce the effectiveness of intraphagocytic brucellacidal mechanisms, possibly contributing to the chronic infection of the udder and its regional LNs. Excretion of *Brucella* into the milk can occur intermittently throughout lactation and may continue during the following pregnancies, reaching considerable numbers (i.e., colony forming units / ml of milk) immediately after delivery and in late lactation (47, 72). Thus, ingestion of contaminated colostrum and milk could be a more frequent route of vertical transmission in small ruminants than *in utero* infection, although lambs born to infected dams may also passively acquire neutralizing antibodies (38, 73, 74). Lambs and kids born from infected females may become latent carriers until sexual maturity; some may develop clinical disease while others remain silently infected (69).

The natural *B. melitensis* infection in non-pregnant small ruminant females is usually asymptomatic (23). Similarly, systemic signs are rare in adult ewes and rams infected by *B. ovis*. Rams with subclinical disease become carriers or shedders of *B. ovis*, and must be diagnosed by culture or serological routine tests and eliminated from the flock.

## Genomic Bases Explain Differences in Pathogenicity and Host Specificity Between *B. melitensis* and *B. ovis*

*B. melitensis* comprises 3 biovars (bvs 1–3), that have similar virulence for goats and sheep. Historically, *B. melitensis* bv 1 is predominant in Latin America, bv 2 in Middle East together with bv 3, which is also more common in European and Africa Mediterranean countries, Eurasia and China, while bvs 1 and 3 seem to be equally present in India (4). Currently, there is only one bv for *B. ovis*. Few comparative genetic studies including both species have been done (Table 2). The genomes of these two *Brucella* species are composed of two circular chromosomes of ~2.1 (ChrI) and 1.2 Mb (ChrII) in size with 3,200–3,300 ORFs. Blasting the genome of *B. melitensis* against that of *B. ovis* reveals a high level of identity at the nucleotide level and almost an 80% of the annotated proteome is shared between them (43). This suggests that the differences in host preference and pathogenesis may be due to a relatively small number of genetic changes and differential mechanisms of gene regulation. Among the differences and probably related with the loss of virulence for most mammalian hosts, it is worth mentioning that *B. ovis* ATCC25840 genome has 264 unique—and 539 missing—annotated protein coding genes as compared to *B. melitensis* 16M genome (43). A comparative whole-genome hybridization study reported that *B. ovis* REO198 genome lacks 80 ORFs distributed in five genomic islands (GI-1, −2, −5, −7, and −9) relative to *B. melitensis* 16M genome (1, 75). Among them, the loss of GI-2 and −5 were proposed as critical for its non-zoonotic nature. GI-2 contains two ORFs that encode for glycosyltransferase enzymes involved in LPS biosynthesis (BME_RS04960 –*wboB*- and BME_RS04965 -*wboA-*) and other ORF encoding for a porin family protein implicated in host cell interaction (BME_RS05010 -Omp25b-). In addition, GI-5 carries homologs of the ABC-type transporters such as Dpp, Opp, and Pot systems, important for attachment to host cell and intracellular survival in other bacteria (1, 75, 76). Contrarily, a highly conserved 26.5 kb GI is present in chromosome II of *B. ovis* strains (*B. ovis* pathogenicity island 1; BOPI-1), but not in *B. melitensis*. This GI contains genes that encoded for an ABC transporter (abcEDCBA), an antitoxin of the toxin-antitoxin system Phd/YefM family (BOV_RS12860), and several pseudogenes and hypothetical proteins (43).

**Table 2:** Comparative genetic studies including *B. melitensis* and *B. ovis*.

**Strains**	**Methods**	***B. ovis* genome with respect to *B. melitensis*.**	**References**
*B. ovis* REO198; *B. melitensis* 16M	Whole-genome hybridization	It lacks 80 ORFs distributed in GIs 1, 2, 5, 7, and 9.	(75)
*B. ovis* ATCC25840; *B. melitensis* 16M	Sequencing of *B. ovis* genome and comparison with reference genomes	Evidences of genome degradation. It contains 264 unique and 539 missing genes.	(43)
*B. melitensis* 16M and ATCC 23457; *B. ovis* ATCC25840	Identification of TCS coding genes in available genomes	There are four inactivated TCS genes (*prlS, tceS, moaR* and *stcA)*	(77)
*B. melitensis* 16M, 63/9 and Ether; *B. ovis* 63/290	DNA microarray	*B. ovis* lacks GIs 1, 2, 6, 11, 13 (*B. melitensis* lacks GI 14; and BME_bv1 also lacks GI15)	(76)

Similar to other host-specific pathogens whose genomes often show signs of genomic decay related to their divergence from a generalist ancestor along with a change in their route of transmission (78), the more restricted tissue tropism (male genital tract) and narrow host range (rams) of *B. ovis* appears to be associated with its genome degradation (1). The higher number of pseudogenes (244 vs. 163) in *B. ovis* ATCC25840 genome with respect to *B. melitensis* 16 M is directly related to the higher number of transposable elements (38 vs. 7) (43). This difference in the number of active insertion sequences favors genomic reduction and consequent loss of virulence (76). Some of the functional *B. melitensis* genes that are pseudogenes in *B. ovis* genome are involved in oral infection ability (*ureC1*), transport and metabolisms of carbohydrates as erythritol (*eryA*) and glucose (*gluP*), cell envelope structure (BME_RS14295, *omp31*) and cytochrome oxidase activity (*ccoO*). Moreover, contrary to *B. melitensis* 16M, *B. ovis* ATCC25840 genome also has four inactivated genes (*prlS, tceS, moaR*, and *stcA*) encoding for protein members of two components systems (TCS), signal transduction mechanisms with important virulence regulatory functions in *Brucella* spp. (77). Indeed, the inactivation of *tceS* and *prlS* may also play a role in the virulence restrictions exhibited by *B. ovis*, since these genes are necessary for the persistence of *B. melitensis* in mice (79, 80).

Despite differences in virulence among *B. melitensis* bvs. have not been reported to date, the absence of a GI-15 in ChrII in *B. melitensis* bv1, compared to bvs. 2 and 3 (76) is notable to mention. GI-15 contains 7 ORFs, which encode homologs to the BRUAB_RS13255 (ex BruAb2_0591; Twin-arginine translocation pathway signal sequence domain-containing protein); BR_RS13010 (Lrp/AsnC family of DNA binding transcriptional regulator), BRUAB_RS13270 and BRUAB_RS13275 (ex BruAb2_0594 and 0595, respectively; ABC transporter substrate-binding proteins) and BRUAB_RS13280 (ex BruAb2_0596; FAD binding oxidoreductase). More research is needed to identify the role of the GI-15 in *B. melitensis* virulence.

## Known and Undefined Mechanisms of *B. melitensis* and *B. ovis* Virulence Factors May Explain Different Pathophysiological Consequences

Both *B. melitensis and B. ovis* show similar ability to invade, survive and replicate inside professional and non-professional phagocytic cells from their natural hosts as well as other hosts (81–83), but their pathophysiological consequences are quite different. Contrary to many bacteria, but similar to other species of the genus *Brucella*, they don't produce or carry any classical virulence factors, such as toxins, pili and or fimbriae, capsule, drug-resistant forms or antigenic variations. Instead, they use several atypical virulence factors to stealthily enter cells, evade intracellular killing and hamper host immune responses (84).

To date, fewer than 200 gene products have been identified as *Brucella* virulence factors (85), although only some of them have been extensively studied, with much more detail in *B. melitensis* than in *B. ovis*. Among them, LPS is the most broadly studied, since its composition modulates a distinctive phenotype between these two *Brucella* species, but it does not seem to affect its virulence. The overall structure of the lipid A and the core oligosaccharide of both LPS species is presumed to be similar, while the lack or the presence of the terminal O-polysaccharide (O-PS) is associated with the rough or smooth phenotypes of the *B. ovis* or *B. melitensis*, respectively. Independently of this major difference, both LPSs are involved in reduced endotoxic activity, low proinflammatory cytokine production, immune system evasion, cell invasion and resistance to complement and antimicrobial peptides destruction, although some of these properties in *B. ovis* are in association with outer membrane protein (OMP) expression pattern (86–90).

OMPs are other well-known virulence factors involved not only in the *Brucella* outer membrane stability but also in the host: pathogen initial interaction and host cell function modulation (91, 92). Among them, a family of highly conserved 25kDa OMPs is one of the most studied in the *Brucella* genus. *B. ovis* Omp25 presents a 36 bp deletion with respect to *B melitensis* that affects a surface-exposed loop of the protein and might influence the *B. ovis*: host interaction (93). Despite this difference, *B. melitensis* and *B. ovis* Omp25 deletion mutants have shown to be attenuated in mice and pregnant goats, and were considered good vaccine candidates (94, 95). Another 25 kDa OMP family difference between these two species of *Brucella* is the absence of the Omp25b encoded gene in the genome of *B. ovis*, although this protein does not seem to be essential for the establishment of Brucella spp. (93). Contrarily, Omp25d and Omp22 play critical roles in the entry of *B. ovis* into mammalian cells, but there is no evidence that they perform this function in *B. melitensis* (82, 96). Many other differences exist between *B. melitensis* and *B. ovis* OMPs and their participation in differential pathogenicity and host preference has been predicted *in silico* or tested *in vitro*, but not by *in vivo* challenge of the preferred host (43, 82, 86, 97, 98).

The two-component system (TCS) *BvrR/BvrS* is an experimentally demonstrated virulence factor in *B. abortus* that not only modulates the homeostasis of the outer membrane but also influence metabolic pathways and *Brucella* adaptation to the intracellular environment (99). The *bvrR*/*S* genes of *B. melitensis* have a high degree of identity with those from *B. abortus* with only four different amino acids in each sequence, and putative *bvrR* and *bvrS* genes exist in *B. ovis* genome (100). While *B. melitensis* BvrR mutants are defective in the invasion of Hela cells (101), *B. ovis* BvrR/S mutants couldn't be generated suggesting that besides the multiple functions attribute to this TCS in smooth *Brucella* strains, would also be necessary for the *in vitro* free survival of *B. ovis* (102).

Similar, VjbR is a global transcriptional activator of virulence factors. Both *B. melitensis* and *B. ovis vjbR* mutants have been reported to have the same level of internalization but may be severely impaired to survive in macrophages and human trophoblastic cell line compared to WT strains (101–104). Consistently, both *vjbR* mutants are cleared from the spleens of infected mice few weeks p.i. (102, 103) and *B. melitensis* Δ*vjbR* is safe in its natural host (36, 105). In *B. melitensis*, VjbR regulates the positive transcription of two of the main virulence factors, the type four secretion system (T4SS) encoded by the *virB* operon, and flagellar genes, during vegetative growth and intracellular survival (101). Apparently, VjbR also regulates the *virB* expression in *B. ovis*, but not flagellar genes (102). Moreover, the flagellar loci seem to be dispensable for *B. ovis* virulence in mice and their expression have not been confirmed to date (106), though some flagellar proteins are expressed in and required for *B. melitensis* virulence in mice and goats (90, 107).

The T4SS is a translocator of effector molecules that allow *Brucella* to survive intracellularly and establishes its replicative niche. Its essential role has been experimentally demonstrated in both *B. ovis and B. melitensis* (90, 108–110), although a different regulatory mechanism was observed. The VirB operon is highly expressed under pH neutral culture conditions in *B ovis* and *B. melitensis* 16M, and is further induced under acidic culture conditions in *B. melitensis* but not in *B. ovis* (111, 112). In addition, the number and identity of the T4SS translocated effectors, the regulation of their expression and the way they modulate the outcome of the infection in both *Brucella* species remains to be elucidated. One example worth mentioning is TcpB (also called BtpA or Btp1), a TIR-domain containing protein, which targets TIRAP and represses pro-inflammatory cytokine production by TLR2- and TLR4-signaling pathways (113). The *tcpB* gene is well-conserved in the *B. ovis* genome (43), but little is known about its expression and function during *B. ovis* infection.

Another potential difference in virulence factors is the function of the cyclic B-1,2-D- glucans (CβGs) in smooth and rough natural *Brucella* species. CβG are polysaccharides present in the periplasmic space of *Brucella* that play an important role in environmental sensing, osmo-adaptation and interaction with the host cell membrane (114). While CβGs-defective *B. ovis* shows less internalization by macrophages, the microorganism's intracellular survival and replication are not affected (102). *B. abortus* mutants of the CβG synthase (*cgs*) are impaired in intracellular replication, but they do not show defects in internalization (115). The different phenotypes could be in part due to CβG contribution to the architecture and stabilization of the bacterial envelope in rough strains, while in smooth strains it may be required in order to avoid the fusion of the lysosomes with the *Brucella*-containing vacuole. At the same time, both *cgs* mutants show an attenuated phenotype in the mouse model of infection (102, 116). Although the participation of CβG in *B. melitensis* molecular pathogenesis has not been studied, it is very likely that its function is homologous to that of *B. abortus*.

Another difference is that *B. ovis* express an ABC transporter system (abcEDCBA) which is absent in *B. melitensis* and that appears to compensate for a lack of alternative nutrient import pathways. This ABC transporter plays a key role in the intra- and extracellular survival of *B. ovis* in cell culture and during the early stages of infection in mice and rams (110, 112, 117).

As we emphasize throughout this section, several structural and functional differences between *B. melitensis'* and *B. ovis'* major virulence factors have been identified, yet few of them have been effectively related to differences in host preference or pathogenesis (Table 3). Undoubtedly, future studies, which consider both the pathogen and the host (cell receptors, molecular mechanisms or environment), will help address these voids of knowledge.

**Table 3:** Comparison of virulence factors in *B. melitensis* and *B. ovis*.

**Virulence factor**	**Descripción**	***B. melitensis***	***B. ovis***
LPS	Structural integrity
Cell invasion and evasion of host immune response
	Similar core and lipid A Similar role in virulence	
		Presence of O-PS	*wboA/B* genes absent
OMPs	Outer membrane stability Interaction with host cells	Many differences in OMP genes Omp25 is involved in virulence in mice	
		Omp25 is involved in virulence in goats	Omp25d and Omp22 are involved in cell internalization
BvrR/BvrS	TCS. Environmental sensing and regulation of gene expression (OMPs, virulence factors)	Essential for cellular invasion	Mutants couldn't be obtained Essential for *in vitro* cell-free survival?
VjbR	Quorum-sensing transcriptional regulator (VirB, flagellar genes)	Involved in intracellular replication and virulence	
T4SS (VirB operon)	Translocator of effector proteins to host cells	Key role in the establishment of *Brucella* replicative niche Essential for intracellular replication and virulence	
		Enhanced expression in acidic conditions *in vitro*	Highest expression at neutral pH
CβG	Environmental sensing
Osmo-adaptation
Interaction with host-cells
	Unknown
Cgs gene conserved with *B. abortus*. Essential for intracellular replication?	Involved in cell internalization and virulence in mice
ABC Transporter abcEDCBA	Unknown
Involved in post-transcriptional regulation of VirB expression	*abcEDCBA* genes absent	Involved in intracellular replication and virulence in mice and rams
Flagellar proteins	Unknown	Essential for virulence in mice and goats	Non-functional
TcpB	T4SS secreted effector	Involved in TLR2/4 inhibition by TIRAP degradation	Unknown Gene conserved
PrlS	Member of TCS PrlS/R (proline sensor–regulator)	Necessary for *Brucella* persistence in mice	Gene absent
TceS	Member of TCS TceS/R	Involved in *Brucella* intracellular survival and virulence in mice.	Gene absent

## Laboratory Procedures for Differential Diagnosis of Brucellosis in Goats and Sheep

Laboratory tests are necessary to differentiate brucellosis in small ruminants from other infectious agents commonly causing abortion (*Campylobacter fetus* subsp. *fetus, Chlamydophila abortus, Toxoplasma gondii, Coxielia burnetti*) and orchio-epididymitis (*Histophilus somni, Actinobacillus seminis*). Laboratory tests include either the detection of immune response or identification of the agent. Bacterial isolation remains the gold standard for diagnosis of brucellosis, but due to several drawbacks previously mentioned, other safer, cheaper, and faster methodologies have been developed. Thus, PCR-based methodologies have successfully replaced culture and allowed further characterization of the agent (Table 4). Because of it practicability and its wide use, conventional PCR has become the primary diagnostic option for specific detection of *B. melitensis* and *B. ovis* DNA in biological samples (118, 119). More recently, a more simple and inexpensive technique called loop-mediated isothermal amplification (LAMP) assay has been optimized for *B. melitensis* DNA detection in samples from goats with clinical disease (120). Molecular techniques also allow: (1) semi-quantification of *Brucella* DNA in biological samples (121), (2) genotyping of *B. melitensis* strains (122), and (3) differentiation between *B. melitensis* field strains and vaccine strain Rev.1 (91, 123).

**Table 4:** PCR-based methods for *B. melitensis* and *B. ovis* specific identification.

**Level of identification**	**Methodology**	**References**
*B. ovis*	Conventional PCR
Multiplex PCR	(119, 124, 125)
*B. melitensis*	Conventional PCR	(126, 127)
	LAMP	(120)
	Real Time (RT)-PCR	(128)
	Immuno-magnetic separation-PCR (IMS-PCR)	(129)
*B. melitensis* biovars	Multiple-locus variable number tandem repeat analysis (MLVA)	(122)
*B. melitensis* field strain and *B. melitensis* Rev.1	PCR-restriction fragment length polymorphism (PCR-RFLP)	(91, 123)
	Duplex PCR	(130)
	Multiplex PCR	(127)
	Single nucleotide polymorphism (SNP)-based test	(131)

Antibody detection tests remain the most cost-effective approach for the screening and detection of *B. melitensis*-infected herds, especially under resource-limited settings. Since no single serological test is adequate in all epidemiological situations, the simultaneous use of at least two different serological techniques that vary according to epidemiological situation is strongly recommended to evaluate brucellosis status in small ruminant herds (14). Macro-agglutination tests using buffered smooth *Brucella* antigen, such as buffered plate agglutination test (BPAT) or rose Bengal test (RBT), or indirect enzyme-linked immunosorbent assays (iELISAs) are generally employed for screening, with fluorescence-polarized antigen (FPA) or complement fixation (CF) as complementary or confirmatory tests, respectively. To confirm false-positive serological results caused by infection with cross-reacting bacteria (i.e., *Y. enterocolitica O:9, E. coli O157 or S. urbana*) in *B. melitensis*-free areas, the brucellin skin test (BST) is the preferred diagnostic test (14, 132–134), and the agar gel immunodiffusion (AGID) test is used to serologically differentiate infected from vaccinated goats and sheep in the field (135). For *B. ovis* serological antibody detection, AGID, ELISA and CF are the recommended techniques; however, they commonly show highly variable results with considerable number of false-positive and false-negative serologic reactions (136). The development of a reliable and easy-to-perform test for serological detection of *B. ovis* will be a necessary step for controlling the disease.

## Prevention and Control Measures

Simple measures to prevent the introduction of the agent in a herd are: (1) to maintain a closed herd, (2) to avoid contact with infected animals and unnecessary visitors, (3) to use separate pens for lambing, and (4) to keep facilities clean and disinfected. If external animals must be incorporated, they should come from an accredited brucellosis-free herd or a herd without history of brucellosis, and the individual serological and clinical negative status must be confirmed before being introduced into the herd. All these measures are easier to implement in intensive farming, but are almost impossible to achieve in extensive, transhumant or nomadic pastoralism situations, which is the way the majority of caprine and ovine production are managed. Thus, surveillance and vaccination, together with imposing livestock movement control, are the main measures aimed at maintaining brucellosis-free flocks in these conditions.

After *B. melitensis* infection, three different options are recommended for control and eradication: (1) test and slaughter of positive animals, (2) massive vaccination, or (3) pre-pubertal female vaccination with test and slaughter of infected animals (18, 137). Although it is not the purpose of this article to describe these options in detail as has been done extensively in previous publications, it is worth noting that much of the success of control and eradication programs is based on reliable laboratory diagnostic results and the use of suitable qualified vaccine lots or seed stock sources used to make the immunogen. The selection of the criteria to be employed depends on, but is not restricted to: (1) the prevalence of brucellosis, (2) the capacities of the National Veterinary Services, (3) the type of animal husbandry, (4) the geography of the area, (5) financial, (6) technical and personnel resources available, and (7) the compliance of the livestock owners, among other issues. On the other hand, the prevalence of *B. ovis* infection can be reduced by the clinical examination and laboratory testing of rams before the breeding season followed by the culling of those with palpable abnormalities or positive laboratory results. Despite some successful experimental attempts, a commercial vaccine to prevent *B. ovis* infection is not yet available (117, 138, 139). The use of antibiotic treatment is not economically or clinically recommended for the control of brucellosis in infected small ruminants, due to the high failure rate, cost and potential problems related to maintaining the coexistence of infected and healthy animals in a flock.

Even though animal vaccination and other measures presented here have contributed significantly to eradicating or reducing the incidence of brucellosis in many geographical areas, the disease has not been controlled in many other regions and is considered, in many cases, a re-emerging disease. A relatively new complementary approach is the identification and subsequent selection of animals with natural resistance to *Brucella* infection. Original studies found naturally resistant pigs and cows (140). Later on, this phenotypic characteristic was linked to genetic markers. The alleles of the goat genes *SLC11A1* (formerly *NRAMP1*), *PTPRT, IRF3*, and *TNF* were recently reported by us associated with the absence of *Brucella* sero-response in Creole crossbreed goats (141–144), and variants in the *MHC-DRB1* loci were associated with brucellosis susceptibility in Chinese Merino sheep (145). Though, it is clear that resistance to natural infection would rarely be controlled by a single gene, more research is still required to include marker-assisted selection for natural resistance to brucellosis in breeding programs as a significant contribution to the prevention of the disease in small ruminant herds.

## Conclusions and Future Approaches

Brucellosis in small ruminants is an ancient disease that has been eradicated in many countries, but still remains endemic in most regions. There is a vast amount of literature explaining successful eradication campaigns in several areas using available tools (i.e., diagnostic tests, vaccines, culling) adjusted to the particular context. Thus, why is small ruminant brucellosis still endemic in some geographic regions? The answer may be that these tools are simply not available, or they are wrongfully used. Undoubtedly, future development of the combination of less complex diagnostic tests, effective immunogens, enhanced response to vaccination, identification of innate resistant animals to *Brucella* infection, and/or other unknown factors will help to control and eradicate brucellosis in domestic sheep and goats. In the meantime, history informs us that control and eradication programs may be successfully implemented employing available tools and appropriate strategies.

## Author Contributions

CR conceived and designed the outline of the review and reviewed and edited the document. CR, EM, and UR performed the literature search and wrote the original draft. All authors read and approved the latest version of the article.

## Funding

This work was supported by the Agencia Nacional de Promoción Científica y Tecnológica de Argentina (Grant # PICT 2018-3811) and I.N.T.A. (Grant # PE-I105).

## Conflict of Interest

The authors declare that the research was conducted in the absence of any commercial or financial relationships that could be construed as a potential conflict of interest.

## Publisher's Note

All claims expressed in this article are solely those of the authors and do not necessarily represent those of their affiliated organizations, or those of the publisher, the editors and the reviewers. Any product that may be evaluated in this article, or claim that may be made by its manufacturer, is not guaranteed or endorsed by the publisher.

## References

[1] Rajendhran J. Genomic insights into Brucella. Infect Genet Evol J Mol Epidemiol Evol Genet Infect Dis. (2021) 87:104635. 10.1016/j.meegid.2020.104635 33189905

[2] Alton GG. Brucella Melitensis. Anim Brucell CRC. Boca Raton, FL: Press Inc. Nielsen K Duncan JR, editors (1990). p. 383–409.

[3] Corbel J. Brucellosis in Humans and Animals. WHO, editor. Geneva: WHO (2006). p. 89.

[4] Rossetti CA Arenas-Gamboa AM Maurizio E. Caprine brucellosis: a historically neglected disease with significant impact on public health. PLoS Negl Trop Dis. (2017) 11:e0005692. 10.1371/journal.pntd.0005692 28817647 PMC5560528

[5] Blasco J. Brucella ovis. In: Nielsen K Duncan JR, editors. Animal Brucellosis. Boca Raton, FL: CRC Press (1990). p. 351–78.

[6] Costa LF Pessoa MS Guimarães LB Faria AKS Morão RP Mol JP da S. Serologic and molecular evidence of *Brucella ovis* infection in ovine and caprine flocks in the State of Minas Gerais, Brazil. BMC Res Notes. (2016) 9:190. 10.1186/s13104-016-1998-2 27017445 PMC4808293

[7] Arnaudov A. Serological survey for Brucella ovis dissemination among goats (*Capra hircus*). J Cent Eur Agric. (2012) 13:188–92. 10.5513/JCEA01/13.1.1033

[8] McCollum M Rhyan J Coburn S Ewalt D Lahr C Nol P. Clinical, culture, serology, and histopathology outcomes of bighorn sheep experimentally infected with *Brucella ovis*. J Wildl Dis. (2013) 49:900–10. 10.7589/2012-02-061 24502717

[9] Ridler AL West DM Stafford KJ Wilson PR Fenwick SG. Transmission of *Brucella ovis* from rams to red deer stags. N Z Vet J. (2000) 48:57–9. 10.1080/00480169.2000.36159 16032119

[10] Garcia Carrillo C Casas Olascoaga R Cuba Caparo A Lucero N Szyfres B. Infección experimental de cabras con *B. ovis*. Estudio bacteriológico, serológico e histopatológico. Rev Arg Microbiol. (1977) 9:101–8. 614651

[11] Burgess GW Spencer TL Norris MJ. Experimental infection of goats with *Brucella ovis*. Aust Vet J. (1985) 62:262–4. 10.1111/j.1751-0813.1985.tb14247.x 4062738

[12] McFarlane D Salisbury R Osborne H Jebson J. Investigation into sheep abortion in New Zealand during 1950 lambing season. Austr Vet J. (1952) 28:221–8. 10.1111/j.1751-0813.1952.tb13477.x

[13] Simmons G Hall W. Epididymitis of rams. Preliminary studies on the occurrence and pathogenicity of a Brucella-like organisms. Austr Vet J. (1953) 29:33–40. 10.1111/j.1751-0813.1953.tb05206.x

[14] OIE. Chapter 3.1.4. In: OIE Terrestrial Manual. Paris, France: OIE (2018). p. 355–98.

[15] Luchsinger DW Anderson RK. Longitudinal studies of naturally acquired *Brucella abortus* infection in sheep. Am J Vet Res. (1979) 40:1307–12. 118693

[16] Ocholi RA Kwaga JKP Ajogi I Bale JOO. Abortion due to *Brucella abortus* in sheep in Nigeria. Rev Sci Tech. (2005) 24:973–9. 10.20506/rst.24.3.1627 16642768

[17] Wareth G Melzer F Tomaso H Roesler U Neubauer H. Detection of *Brucella abortus* DNA in aborted goats and sheep in Egypt by real-time PCR. BMC Res Notes. (2015) 8:212. 10.1186/s13104-015-1173-1 26036697 PMC4467612

[18] Alton GG. Control of *Brucella melitensis* infection in sheep and goats–a review. Trop Anim Health Prod. (1987) 19:65–74. 10.1007/BF02297320 3307078

[19] Cao X Li Z Liu Z Fu B Liu Y Shang Y. Molecular epidemiological characterization of Brucella isolates from sheep and yaks in northwest China. Transbound Emerg Dis. (2018) 65:e425–33. 10.1111/tbed.12777 29193808

[20] Lucero NE Ayala SM Escobar GI Jacob NR. Brucella isolated in humans and animals in Latin America from 1968 to 2006. Epidemiol Infect. (2008) 136:496–503. 10.1017/S0950268807008795 17559694 PMC2870831

[21] Paolicchi FA Terzolo HR Campero CM. Isolation of *Brucella suis* from the semen of a ram. Vet Rec. (1993) 132:67. 10.1136/vr.132.3.67 8430486

[22] Qing D. Isolation of *Brucella suis* from cattle and sheep. Chinese J Vet Med. (1993) 19:23–4.

[23] Mazlina M Khairani-Bejo S Hazilawati H Shaqinah NN Zamri-Saad M. Antigenic distribution, pathological changes, antibody response and serological detection in non-pregnant goats following experimental infection by *Brucella melitensis*. Transbound Emerg Dis. (2021) 68:2028–38. 10.1111/tbed.13850 32979887

[24] Petrović M Špičić S Potkonjak A Lako B Kostov M Cvetnić Ž. First evidence of *Brucella ovis* infection in rams in the Pirot Municipality, Serbia. Vet Ital. (2014) 50:259–68. 25546063 10.12834/VetIt.1305.09.11

[25] Alton G Jones L Angus R Verger J. Techniques for the Brucellosis Laboratory. 3rd ed. Paris: INRA (1988). p. 190.

[26] Pérez-Etayo L de Miguel MJ Conde-Álvarez R Muñoz PM Khames M Iriarte M. The CO(2)-dependence of *Brucella ovis* and *Brucella abortus* biovars is caused by defective carbonic anhydrases. Vet Res. (2018) 49:85. 10.1186/s13567-018-0583-1 30185220 PMC6126018

[27] Meyer ME. Phenotypic comparison of Brucella ovis to the DNA-homologous Brucella species. Am J Vet Res. (1969) 30:1757–64. 5824903

[28] Marín CM Alabart JL Blasco JM. Effect of antibiotics contained in two Brucella selective media on growth of *Brucella abortus, B. melitensis*, and *B. ovis*. J Clin Microbiol. (1996) 34:426–8. 10.1128/jcm.34.2.426-428.1996 8789029 PMC228811

[29] Terzolo HR Paolicchi FA Moreira AR Homse A. Skirrow agar for simultaneous isolation of Brucella and Campylobacter species. Vet Rec. (1991) 129:531–2. 1788919

[30] De Miguel MJ Marín CM Muñoz PM Dieste L Grilló MJ Blasco JM. Development of a selective culture medium for primary isolation of the main Brucella species. J Clin Microbiol. (2011) 49:1458–63. 10.1128/JCM.02301-10 21270216 PMC3122841

[31] Anonymous. Brucellosis in sheep and goats (*B. melitensis*). European Scientific Committee on Animal Health & Welfare. Opinions. (2001). p. 89. Available online at: https://ec.europa.eu/food/system/files/2020-12/sci-com_scah_out59_en.pdf

[32] von Bargen K Gagnaire A Arce-Gorvel V de Bovis B Baudimont F Chasson L. Cervical lymph nodes as a selective niche for Brucella during oral infections. PLoS ONE. (2014) 10:e0121790. 10.1371/journal.pone.0121790 25919005 PMC4412401

[33] Rossetti CA Drake KL Siddavatam P Lawhon SD Nunes JES Gull T. Systems biology analysis of Brucella infected Peyer's patch reveals rapid invasion with modest transient perturbations of the host transcriptome. PLoS ONE. (2013) 8:e81719. 10.1371/journal.pone.0081719 24349118 PMC3857238

[34] Byndloss MX Tsai AY Walker GT Miller CN Young BM English BC. *Brucella abortus* infection of placental trophoblasts triggers endoplasmic reticulum stress-mediated cell death and fetal loss via type IV secretion system-dependent activation of CHOP. mBio. (2019) 10:e01538-19. 10.1128/mBio.01538-19 31337727 PMC6650558

[35] Alexander B Schnurrenberger PR Brown RR. Numbers of *Brucella abortus* in the placenta, umbilicus and fetal fluid of two naturally infected cows. Vet Rec. (1981) 108:500. 10.1136/vr.108.23.500 6795789

[36] Hensel ME Garcia-Gonzalez DG Chaki SP Hartwig A Gordy PW Bowen R. Vaccine candidate *Brucella melitensis* 16MΔvjbR is safe in a pregnant sheep model and confers protection. mSphere. (2020) 5:e00120-20. 10.1128/mSphere.00120-20 32404509 PMC7227765

[37] Pappas G Panagopoulou P Christou L Akritidis N. Brucella as a biological weapon. Cell Mol Life Sci. (2006) 63:2229–36. 10.1007/s00018-006-6311-4 16964579 PMC11136069

[38] Buddle M. Observations on the transmission of Brucella infection in sheep. N Z Vet J. (1955) 3:10–9. 10.1080/00480169.1955.331723557828

[39] Blasco JM Barberan M. Brucelosis. Epidemiologia, patogenia y cuadro clinico. Ovis. (1990) 8:25–32.

[40] Elderbrook M Schumaker B Cornish T Peck D Sondgeroth K. Seroprevalence and risk factors of Brucella ovis in domestic sheep in Wyoming, USA. BMC Vet Res. (2019) 15:246. 10.1186/s12917-019-1995-5 31307483 PMC6631759

[41] Sergeant ES. Seroprevalence of *Brucella ovis* infection in commercial ram flocks in the Tamworth area. N Z Vet J. (1994) 42:97–100. 10.1080/00480169.1994.35795 16031755

[42] Bulgin MS. Epididymitis in rams and lambs. Vet Clin North Am Food Anim Pract. (1990) 6:683–90. 10.1016/S0749-0720(15)30840-9 2245369

[43] Tsolis RM Seshadri R Santos RL Sangari FJ Lobo JMG de Jong MF. Genome degradation in *Brucella ovis* corresponds with narrowing of its host range and tissue tropism. PLoS ONE. (2009) 4:e5519. 10.1371/journal.pone.0005519 19436743 PMC2677664

[44] Ridler AL West DM Stafford KJ Wilson PR. Persistence, serodiagnosis and effects on semen characteristics of artificial *Brucella ovis* infection in red deer stags. N Z Vet J. (2006) 54:85–90. 10.1080/00480169.2006.36617 16596160

[45] Hartley W Lebson J McFarlane D. Some observations on natural transmission of ovine brucellosis. N Z Vet J. (1955) 3:5–10. 10.1080/00480169.1955.33171

[46] EFSA Panel on animal health and welfare EA. Ovine epididymitis (*Brucella ovis*). EFSA J. (2017) 15:4994. 10.2903/j.efsa.2017.4994 32625291 PMC7010007

[47] Grilló MJ Marín CM Barberán M Blasco JM. Experimental *Brucella ovis* infection in pregnant ewes. Vet Rec. (1999) 144:555–8. 10.1136/vr.144.20.555 10371013

[48] Ris DR. The bacteriology and serology of ewes inoculated with viable *Brucella ovis* organisms. N Z Vet J. (1970) 18:2–7. 10.1080/00480169.1970.33846 5266508

[49] Marco J González L Cuervo LA Beltrán de Heredia F Barberán M Marín C. Brucella ovis infection in two flocks of sheep. Vet Rec. (1994) 135:254–6. 10.1136/vr.135.11.254 7810048

[50] Igwebuike UM. Trophoblast cells of ruminant placentas–a minireview. Anim Reprod Sci. (2006) 93:185–98. 10.1016/j.anireprosci.2005.06.003 16043315

[51] Samartino LE Enright FM. *Brucella abortus* differs in the multiplication within bovine chorioallantoic membrane explants from early and late gestation. Comp Immunol Microbiol Infect Dis. (1996) 19:55–63. 10.1016/0147-9571(95)00018-6 8654046

[52] Wang X Lin P Li Y Xiang C Yin Y Chen Z. *Brucella suis* vaccine strain 2 induces endoplasmic reticulum stress that affects intracellular replication in goat trophoblast cells *in vitro*. Front Cell Infect Microbiol. (2016) 6:19. 10.3389/fcimb.2016.00019 26904517 PMC4746994

[53] Carvalho Neta AV Stynen APR Paixão TA Miranda KL Silva FL Roux CM. Modulation of the bovine trophoblastic innate immune response by *Brucella abortus*. Infect Immun. (2008) 76:1897–907. 10.1128/IAI.01554-07 18316388 PMC2346690

[54] Fernández AG Ferrero MC Hielpos MS Fossati CA Baldi PC. Proinflammatory response of human trophoblastic cells to *Brucella abortus* infection and upon interactions with infected phagocytes. Biol Reprod. (2016) 94:48. 10.1095/biolreprod.115.131706 26792938

[55] García-Méndez KB Hielpos SM Soler-Llorens PF Arce-Gorvel V Hale C Gorvel J-P. Infection by *Brucella melitensis* or *Brucella papionis* modifies essential physiological functions of human trophoblasts. Cell Microbiol. (2019) 21:e13019. 10.1111/cmi.13019 30817085

[56] Anderson TD Meador VP Cheville NF. Pathogenesis of placentitis in the goat inoculated with *Brucella abortus*. I. Gross and histologic lesions. Vet Pathol. (1986) 23:219–26. 10.1177/030098588602300301 3088809

[57] Anderson TD Cheville NF Meador VP. Pathogenesis of placentitis in the goat inoculated with *Brucella abortus*. II. Ultrastructural studies. Vet Pathol. (1986) 23:227–39. 10.1177/030098588602300302 3088810

[58] Petersen E Rajashekara G Sanakkayala N Eskra L Harms J Splitter G. Erythritol triggers expression of virulence traits in *Brucella melitensis*. Microbes Infect. (2013) 15:440–9. 10.1016/j.micinf.2013.02.002 23421980 PMC3686989

[59] Inan A Erdem H Elaldi N Gulsun S Karahocagil MK Pekok AU. Brucellosis in pregnancy: results of multicenter ID-IRI study. Eur J Clin Microbiol Infect Dis Off Publ Eur Soc Clin Microbiol. (2019) 38:1261–8. 10.1007/s10096-019-03540-z 30989418

[60] Salcedo SP Chevrier N Lacerda TLS Ben Amara A Gerart S Gorvel VA. Pathogenic brucellae replicate in human trophoblasts. J Infect Dis. (2013) 207:1075–83. 10.1093/infdis/jit007 23303808

[61] Hensel ME Chaki SP Stranahan L Gregory AE van Schaik EJ Garcia-Gonzalez DG. Intratracheal inoculation with *Brucella melitensis* in the pregnant guinea pig is an improved model for reproductive pathogenesis and vaccine studies. Infect Immun. (2020) 88:e00204-20. 10.1128/IAI.00204-20 32690632 PMC7504952

[62] Wang Z Wang SS Wang GL Wu TL Lv YL Wu QM. A pregnant mouse model for the vertical transmission of *Brucella melitensis*. Vet J. (2014) 200:116–21. 10.1016/j.tvjl.2013.12.021 24462801

[63] Barbier T Machelart A Zúñiga-Ripa A Plovier H Hougardy C Lobet E. Erythritol availability in bovine, murine and human models highlights a potential role for the host aldose reductase during Brucella infection. Front Microbiol. (2017) 8:1088. 10.3389/fmicb.2017.01088 28659902 PMC5468441

[64] Carvalho Junior C Moustacas V Xavier N Costa E Costa L Silva T. Andrological, pathologic, morphometric and ultrasonographic findings in rams experimentally infected with *Brucella ovis*. Small Rum Res. (2012) 102:213–22. 10.1016/j.smallrumres.2011.08.004

[65] Burgess GW. Ovine contagious epididymitis: a review. Vet Microbiol. (1982) 7:551–75. 10.1016/0378-1135(82)90049-9 6762755

[66] Ahmad R Niaz B. Orchitis due to brucellosis in a buck. Pakistan Vet J. (1998) 18:146–9.

[67] Ali A Derar DR Osman SA Tharwat M Al-Sobayil F Elshahed M. Scrotal enlargement in rams and bucks in Qassim region, central of Saudi Arabia: clinical and ultrasonographic findings and seroprevalence of brucellosis. Trop Anim Health Prod. (2019) 51:2109–14. 10.1007/s11250-019-01937-8 31161484

[68] Chand P Sadana JR Malhotra AK. Epididymo-orchitis caused by *Brucella melitensis* in breeding rams in India. Vet Rec. (2002) 150:84–5. 10.1136/vr.150.3.84 11837594

[69] Diaz Aparicio E. Epidemiology of brucellosis in domestic animals caused by *Brucella melitensis, Brucella suis* and *Brucella abortus*. Rev Sci Tech. (2013) 32:53–60. 10.20506/rst.32.1.2187 23837364

[70] Meador VP Deyoe BL Cheville NF. Pathogenesis of *Brucella abortus* infection of the mammary gland and supramammary lymph node of the goat. Vet Pathol. (1989) 26:357–68. 10.1177/030098588902600501 2511656

[71] Meador VP Deyoe BL Cheville NF. Effect of nursing on *Brucella abortus* infection of mammary glands of goats. Vet Pathol. (1989) 26:369–75. 10.1177/030098588902600502 2511657

[72] Morgan W McDiarmid A. The excretion of B. abortus in the milk of experimentally infected cattle. Res Vet Sci. (1960) 1:53–6. 10.1016/S0034-5288(18)35029-X

[73] Keogh J Doolette J Clapp K. The epidemiology of ovine brucellosis in South Australia. Aust Vet J. (1958) 34:412–7. 10.1111/j.1751-0813.1958.tb05812.x

[74] Clapp K Keogh J Richards M. Epidemiology of ovine brucellosis in South Australia. Aust Vet J. (1962) 38:482–6. 10.1111/j.1751-0813.1962.tb03995.x

[75] Rajashekara G Glasner JD Glover DA Splitter GA. Comparative whole-genome hybridization reveals genomic islands in Brucella species. J Bacteriol. (2004) 186:5040–51. 10.1128/JB.186.15.5040-5051.2004 15262941 PMC451633

[76] Zhong Z Wang Y Xu J Chen Y Ke Y Zhou X. Parallel gene loss and acquisition among strains of different Brucella species and biovars. J Microbiol. (2012) 50:567–74. 10.1007/s12275-012-2022-8 22923103

[77] Lavín JL Binnewies TT Pisabarro AG Ussery DW García-Lobo JM Oguiza JA. Differences in two-component signal transduction proteins among the genus Brucella: implications for host preference and pathogenesis. Vet Microbiol. (2010) 144:478–83. 10.1016/j.vetmic.2010.01.007 20153589

[78] Bäumler A Fang FC. Host specificity of bacterial pathogens. Cold Spring Harb Perspect Med. (2013) 3:a010041. 10.1101/cshperspect.a010041 24296346 PMC3839602

[79] Mirabella A Yañez Villanueva R-M Delrue R-M Uzureau S Zygmunt MS Cloeckaert A. The two-component system PrlS/PrlR of Brucella melitensis is required for persistence in mice and appears to respond to ionic strength. Microbiology. (2012) 158(Pt 10):2642–51. 10.1099/mic.0.060863-0 22859617

[80] Li Z Fu Q Wang Z Li T Zhang H Guo F. TceSR two-component regulatory system of Brucella melitensis 16M is involved in invasion, intracellular survival and regulated cytotoxicity for macrophages. Lett Appl Microbiol. (2015) 60:565–71. 10.1111/lam.12408 25721466

[81] He Y Reichow S Ramamoorthy S Ding X Lathigra R Craig JC. Brucella melitensis triggers time-dependent modulation of apoptosis and down-regulation of mitochondrion-associated gene expression in mouse macrophages. Infect Immun. (2006) 74:5035–46. 10.1128/IAI.01998-05 16926395 PMC1594834

[82] Martín-Martín AI Caro-Hernández P Orduña A Vizcaíno N Fernández-Lago L. Importance of the Omp25/Omp31 family in the internalization and intracellular replication of virulent B. ovis in murine macrophages and HeLa cells. Microbes Infect. (2008) 10:706–10. 10.1016/j.micinf.2008.02.013 18457973

[83] Rossetti CA Drake KL Adams LG. Transcriptome analysis of HeLa cells response to *Brucella melitensis* infection: a molecular approach to understand the role of the mucosal epithelium in the onset of the Brucella pathogenesis. Microbes Infect. (2012) 14:756–67. 10.1016/j.micinf.2012.03.003 22484383 PMC3389182

[84] De Figueiredo P Ficht TA Rice-Ficht A Rossetti CA Adams LG. Pathogenesis and immunobiology of brucellosis: review of Brucella-host interactions. Am J Pathol. (2015) 185:1505–17. 10.1016/j.ajpath.2015.03.003 25892682 PMC4450313

[85] He Y. Analyses of Brucella pathogenesis, host immunity, and vaccine targets using systems biology and bioinformatics. Front Cell Infect Microbiol. (2012) 2:2. 10.3389/fcimb.2012.00002 22919594 PMC3417401

[86] Caro-Hernández P Fernández-Lago L de Miguel M-J Martín-Martín AI Cloeckaert A Grilló M-J. Role of the Omp25/Omp31 family in outer membrane properties and virulence of *Brucella ovis*. Infect Immun. (2007) 75:4050–61. 10.1128/IAI.00486-07 17562767 PMC1952020

[87] Martín-Martín AI Sancho P Tejedor C Fernández-Lago L Vizcaíno N. Differences in the outer membrane-related properties of the six classical Brucella species. Vet J. (2011) 189:103–5. 10.1016/j.tvjl.2010.05.021 20576453

[88] Moreno E Jones LM Berman DT. Immunochemical characterization of rough *Brucella lipopolysaccharides*. Infect Immun. (1984) 43:779–82. 10.1128/iai.43.3.779-782.1984 6421737 PMC264247

[89] Tumurkhuu G Koide N Takahashi K Hassan F Islam S Ito H. Characterization of biological activities of *Brucella melitensis* lipopolysaccharide. Microbiol Immunol. (2006) 50:421–7. 10.1111/j.1348-0421.2006.tb03810.x 16785713

[90] Zygmunt MS Hagius SD Walker JV Elzer PH. Identification of *Brucella melitensis* 16M genes required for bacterial survival in the caprine host. Microbes Infect. (2006) 8:2849–54. 10.1016/j.micinf.2006.09.002 17090391

[91] Cloeckaert A Vizcaíno N Paquet J-Y Bowden RA Elzer PH. Major outer membrane proteins of *Brucella* spp.: past, present and future. Vet Microbiol. (2002) 90:229–47. 10.1016/S0378-1135(02)00211-0 12414146

[92] Roop RM II Barton IS Hopersberger D Martin DW. Uncovering the hidden credentials of brucella virulence. Microbiol Mol Biol Rev. (2021) 85:e00021-19. 10.1128/MMBR.00021-19 33568459 PMC8549849

[93] Vizcaíno N Caro-Hernández P Cloeckaert A Fernández-Lago L. DNA polymorphism in the omp25/omp31 family of *Brucella* spp.: identification of a 1.7-kb inversion in Brucella cetaceae and of a 15.1-kb genomic island, absent from Brucella ovis, related to the synthesis of smooth lipopolysaccharide. Microbes Infect. (2004) 6:821–34. 10.1016/j.micinf.2004.04.009 15374004

[94] Edmonds MD Cloeckaert A Hagius SD Samartino LE Fulton WT Walker JV. Pathogenicity and protective activity in pregnant goats of a *Brucella melitensis* Deltaomp25 deletion mutant. Res Vet Sci. (2002) 72:235–9. 10.1053/rvsc.2002.0555 12076120

[95] Edmonds MD Cloeckaert A Elzer PH. Brucella species lacking the major outer membrane protein Omp25 are attenuated in mice and protect against *Brucella melitensis* and *Brucella ovis*. Vet Microbiol. (2002) 88:205–21. 10.1016/S0378-1135(02)00110-4 12151196

[96] Manterola L Guzmán-Verri C Chaves-Olarte E Barquero-Calvo E de Miguel M-J Moriyón I. BvrR/BvrS-controlled outer membrane proteins Omp3a and Omp3b are not essential for *Brucella abortus* virulence. Infect Immun. (2007) 75:4867–74. 10.1128/IAI.00439-07 17664262 PMC2044513

[97] Paci V Krasteva I Orsini M Di Febo T Luciani M Perletta F. Proteomic analysis of *Brucella melitensis* and *Brucella ovis* for identification of virulence factor using bioinformatics approachs. Mol Cell Probes. (2020) 53:101581. 10.1016/j.mcp.2020.101581 32428653

[98] Sidhu-Muñoz RS Sancho P Vizcaíno N. *Brucella ovis* PA mutants for outer membrane proteins Omp10, Omp19, SP41, and BepC are not altered in their virulence and outer membrane properties. Vet Microbiol. (2016) 186:59–66. 10.1016/j.vetmic.2016.02.010 27016758

[99] Viadas C Rodríguez MC Sangari FJ Gorvel J-P García-Lobo JM López-Goñi I. Transcriptome analysis of the *Brucella abortus* BvrR/BvrS two-component regulatory system. PLoS ONE. (2010) 5:e10216. 10.1371/journal.pone.0010216 20422049 PMC2858072

[100] López-Goñi I Guzmán-Verri C Manterola L Sola-Landa A Moriyón I Moreno E. Regulation of Brucella virulence by the two-component system BvrR/BvrS. Vet Microbiol. (2002) 90:329–39. 10.1016/S0378-1135(02)00218-3 12414153

[101] Delrue R-M Deschamps C Léonard S Nijskens C Danese I Schaus J-M. A quorum-sensing regulator controls expression of both the type IV secretion system and the flagellar apparatus of *Brucella melitensis*. Cell Microbiol. (2005) 7:1151–61. 10.1111/j.1462-5822.2005.00543.x 16008582

[102] Martín-Martín AI Sancho P de Miguel MJ Fernández-Lago L Vizcaíno N. Quorum-sensing and BvrR/BvrS regulation, the type IV secretion system, cyclic glucans, and BacA in the virulence of *Brucella ovis*: similarities to and differences from smooth brucellae. Infect Immun. (2012) 80:1783–93. 10.1128/IAI.06257-11 22392933 PMC3347429

[103] Arenas-Gamboa AM Ficht TA Kahl-McDonagh MM Rice-Ficht AC. Immunization with a single dose of a microencapsulated *Brucella melitensis* mutant enhances protection against wild-type challenge. Infect Immun. (2008) 76:2448–55. 10.1128/IAI.00767-07 18362129 PMC2423109

[104] Sidhu-Muñoz RS Sancho P Vizcaíno N. Evaluation of human trophoblasts and ovine testis cell lines for the study of the intracellular pathogen *Brucella ovis*. FEMS Microbiol Lett. (2018) 365:1–9. 10.1093/femsle/fny278 30476113

[105] Castaño-Zubieta MR Rossetti CA Maurizio E Hensel ME Arenas-Gamboa ÁM. Evaluation of the safety profile of the vaccine candidate *Brucella melitensis* 16MΔvjbR strain in goats. Vaccine. (2021) 39:617–25. 10.1016/j.vaccine.2020.11.033 33328142 PMC8730362

[106] Sidhu-Muñoz RS Tejedor C Vizcaíno N. The Three Flagellar loci of *Brucella ovis* PA are dispensable for virulence in cellular models and mice. Front Vet Sci. (2020) 7:441. 10.3389/fvets.2020.00441 32851024 PMC7410920

[107] Fretin D Fauconnier A Köhler S Halling S Léonard S Nijskens C. The sheathed flagellum of *Brucella melitensis* is involved in persistence in a murine model of infection. Cell Microbiol. (2005) 7:687–98. 10.1111/j.1462-5822.2005.00502.x 15839898

[108] Delrue RM Martinez-Lorenzo M Lestrate P Danese I Bielarz V Mertens P. Identification of *Brucella* spp. genes involved in intracellular trafficking. Cell Microbiol. (2001) 3:487–97. 10.1046/j.1462-5822.2001.00131.x 11437834

[109] Sá JC Silva TMA Costa EA Silva APC Tsolis RM Paixão TA. The virB-encoded type IV secretion system is critical for establishment of infection and persistence of *Brucella ovis* infection in mice. Vet Microbiol. (2012) 159:130–40. 10.1016/j.vetmic.2012.03.029 22483850

[110] Macedo AA Silva APC Mol JPS Costa LF Garcia LNN Araújo MS. The abcEDCBA-encoded ABC transporter and the virB operon-encoded type IV secretion system of *Brucella ovis* are critical for intracellular trafficking and survival in ovine monocyte-derived macrophages. PLoS ONE. (2015) 10:e0138131. 10.1371/journal.pone.0138131 26366863 PMC4569489

[111] Rouot B Alvarez-Martinez M-T Marius C Menanteau P Guilloteau L Boigegrain R-A. Production of the type IV secretion system differs among Brucella species as revealed with VirB5- and VirB8-specific antisera. Infect Immun. (2003) 71:1075–82. 10.1128/IAI.71.3.1075-1082.2003 12595417 PMC148853

[112] Silva TMA Mol JPS Winter MG Atluri V Xavier MN Pires SF. The predicted ABC transporter AbcEDCBA is required for type IV secretion system expression and lysosomal evasion by *Brucella ovis*. PLoS ONE. (2014) 9:e114532. 10.1371/journal.pone.0114532 25474545 PMC4256435

[113] Radhakrishnan GK Yu Q Harms JS Splitter GA. Brucella TIR domain-containing protein mimics properties of the toll-like receptor adaptor protein TIRAP. J Biol Chem. (2009) 284:9892–8. 10.1074/jbc.M805458200 19196716 PMC2665112

[114] Guidolin LS Arce-Gorvel V Ciocchini AE Comerci DJ Gorvel J-P. Cyclic β-glucans at the bacteria-host cells interphase: one sugar ring to rule them all. Cell Microbiol. (2018) 20:e12850. 10.1111/cmi.12850 29624823

[115] Arellano-Reynoso B Lapaque N Salcedo S Briones G Ciocchini AE Ugalde R. Cyclic beta-1,2-glucan is a Brucella virulence factor required for intracellular survival. Nat Immunol. (2005) 6:618–25. 10.1038/ni1202 15880113

[116] Briones G Iñón de Iannino N Roset M Vigliocco A Paulo PS Ugalde RA. Brucella abortus cyclic beta-1,2-glucan mutants have reduced virulence in mice and are defective in intracellular replication in HeLa cells. Infect Immun. (2001) 69:4528–35. 10.1128/IAI.69.7.4528-4535.2001 11401996 PMC98529

[117] Silva APC Macêdo AA Costa LF Rocha CE Garcia LNN Farias JRD. Encapsulated *Brucella ovis* lacking a putative ATP-binding cassette transporter (ΔabcBA) protects against wild type *Brucella ovis* in rams. PLoS ONE. (2015) 10:e0136865. 10.1371/journal.pone.0136865 26317399 PMC4552948

[118] Leyla G Kadri G Umran O. Comparison of polymerase chain reaction and bacteriological culture for the diagnosis of sheep brucellosis using aborted fetus samples. Vet Microbiol. (2003) 93:53–61. 10.1016/S0378-1135(02)00442-X 12591206

[119] Xavier MN Silva TMA Costa EA Paixão TA Moustacas VS Carvalho CAJ. Development and evaluation of a species-specific PCR assay for the detection of *Brucella ovis* infection in rams. Vet Microbiol. (2010) 145:158–64. 10.1016/j.vetmic.2010.02.037 20347534

[120] Saini S Gupta VK Gururaj K Singh DD Pawaiya RVS Gangwar NK. Comparative diagnostic evaluation of OMP31 gene based TaqMan® real-time PCR assay with visual LAMP assay and indirect ELISA for caprine brucellosis. Trop Anim Health Prod. (2017) 49:1253–64. 10.1007/s11250-017-1323-7 28638960

[121] Hinić V Brodard I Thomann A Cvetnić Z Makaya PV Frey J. Novel identification and differentiation of *Brucella melitensis, B. abortus, B. suis, B. ovis, B. canis*, and *B. neotomae* suitable for both conventional and real-time PCR systems. J Microbiol Methods. (2008) 75:375–8. 10.1016/j.mimet.2008.07.002 18675856

[122] Bricker BJ Ewalt DR Halling SM. Brucella “HOOF-Prints”: strain typing by multi-locus analysis of variable number tandem repeats (VNTRs). BMC Microbiol. (2003) 3:15. 10.1186/1471-2180-3-15 12857351 PMC183870

[123] Bardenstein S Mandelboim M Ficht TA Baum M Banai M. Identification of the Brucella melitensis vaccine strain Rev.1 in animals and humans in Israel by PCR analysis of the PstI site polymorphism of its omp2 gene. J Clin Microbiol. (2002) 40:1475–80. 10.1128/JCM.40.2.1475-1480.2002 11923376 PMC140367

[124] Saunders VF Reddacliff LA Berg T Hornitzky M. Multiplex PCR for the detection of Brucella ovis, Actinobacillus seminis and Histophilus somni in ram semen. Aust Vet J. (2007) 85:72–7. 17300467 10.1111/j.1751-0813.2006.00098.x

[125] Moustacas VS Silva TMA Costa LF Xavier MN Carvalho CAJ Costa ÉA. Species-specific multiplex PCR for the diagnosis of Brucella ovis, Actinobacillus seminis, and Histophilus somni infection in rams. BMC Vet Res. (2013) 9:51. 10.1186/1746-6148-9-51 23514236 PMC3614447

[126] Bricker BJ Halling SM. Differentiation of Brucella abortus bv. 1, 2, and 4, Brucella melitensis, Brucella ovis, and Brucella suis bv. 1 by PCR. J Clin Microbiol. (1994) 32:2660–6. 7852552 10.1128/jcm.32.11.2660-2666.1994 PMC264138

[127] García-Yoldi D Marín CM de Miguel MJ Muñoz PM Vizmanos JL López-Goñi I. Multiplex PCR assay for the identification and differentiation of all Brucella species and the vaccine strains Brucella abortus S19 and RB51 and Brucella melitensis Rev1. Clin Chem. (2006) 52:779–81. 16595839 10.1373/clinchem.2005.062596

[128] Redkar R Rose S Bricker B DelVecchio V. Real-time detection of Brucella abortus, Brucella melitensis and Brucella suis. Mol Cell Probes. (2001) 15:43–52. 11162079 10.1006/mcpr.2000.0338

[129] Ongör H Cetinkaya B Karahan M Bulut H. Evaluation of immunomagnetic separation-polymerase chain reaction in direct detection of Brucella abortus and Brucella melitensis from cheese samples. Foodborne Pathog Dis. (2006) 3:245–50. 16972772 10.1089/fpd.2006.3.245

[130] Alvarez LP García-Effrón G Robles CA. Identification of Brucella ovis exclusive genes in field isolates from Argentina. Vet J. (2016) 209:196–8. 26831160 10.1016/j.tvjl.2015.12.005

[131] Gopaul KK Sells J Bricker BJ Crasta OR Whatmore AM. Rapid and reliable single nucleotide polymorphism-based differentiation of Brucella live vaccine strains from field strains. J Clin Microbiol. (2010) 48:1461–4. 20181906 10.1128/JCM.02193-09 PMC2849582

[132] Bonfini B Chiarenza G Paci V Sacchini F Salini R Vesco G. Cross-reactivity in serological tests for brucellosis: a comparison of immune response of *Escherichia coli* O157:H7 and *Yersinia enterocolitica* O:9 vs *Brucella* spp. Vet Ital. (2018) 54:107–14. 10.12834/VetIt.1176.6539.2 30019327

[133] Chenais E Bagge E Lambertz ST Artursson K. Yersinia enterocolitica serotype O:9 cultured from Swedish sheep showing serologically false-positive reactions for *Brucella melitensis*. Infect Ecol Epidemiol. (2012) 2:19027. 10.3402/iee.v2i0.19027 23240071 PMC3521102

[134] Nielsen K Smith P Yu WL Halbert G. *Salmonella enterica* serotype urbana interference with brucellosis serology. J Immunoassay Immunochem. (2007) 28:289–96. 10.1080/15321810701454904 17613674

[135] Erdenebaatar J Sugar S Yondondorj A Nagabayashi T Syuto B Watarai M. Serological differentiation of Brucella-vaccinated and -infected domesticated animals by the agar gel immunodiffusion test using Brucella polysaccharide in mongolia. J Vet Med Sci. (2002) 64:839–41. 10.1292/jvms.64.839 12399611

[136] Barreto JVP Oliveira PAM Pertile SFN Sbizera MCR Rego FCA Queiroz GR. Non-agreement between 2 serologic techniques for detecting antibody to Brucella ovis in naturally infected sheep. J Vet diagnostic Investig Off Publ Am Assoc Vet Lab Diagnosticians Inc. (2022) 34:164–6. 10.1177/10406387211053589 34697960 PMC8689018

[137] Blasco JM Molina-Flores B. Control and eradication of *Brucella melitensis* infection in sheep and goats. Vet Clin North Am Food Anim Pract. (2011) 27:95–104. 10.1016/j.cvfa.2010.10.003 21215893

[138] Da Costa Martins R Irache JM Blasco JM Muñoz MP Marín CM Jesús Grilló M. Evaluation of particulate acellular vaccines against *Brucella ovis* infection in rams. Vaccine. (2010) 28:3038–46. 10.1016/j.vaccine.2009.10.073 19887131

[139] Díaz AG Quinteros DA Paolicchi FA Rivero MA Palma SD Pardo RP. Mucosal immunization with polymeric antigen BLSOmp31 using alternative delivery systems against Brucella ovis in rams. Vet Immunol Immunopathol. (2019) 209:70–7. 10.1016/j.vetimm.2019.02.005 30885309

[140] Templeton J Adams L. Natural resistance to bovine brucellosis. In: Adams L, editor. Advance in Brucellosis Research: An International Symposium. College Station, TX: Texas A&M University Press (1990) p. 144–50.

[141] Iacoboni PA Hasenauer FC Caffaro ME Gaido A Rossetto C Neumann RD. Polymorphisms at the 3' untranslated region of SLC11A1 gene are associated with protection to Brucella infection in goats. Vet Immunol Immunopathol. (2014) 160:230–234. 10.1016/j.vetimm.2014.05.007 24906349

[142] Rossi UA Hasenauer FC Caffaro ME Neumann R Salatin A Poli MA. A haplotype at intron 8 of PTPRT gene is associated with resistance to Brucella infection in *Argentinian creole* goats. Vet Microbiol. (2017) 207:133–7. 10.1016/j.vetmic.2017.06.001 28757013

[143] Rossi UA Hasenauer FC Caffaro ME Raschia MA Maurizio E Cortez HS. Association of an IRF3 putative functional uORF variant with resistance to Brucella infection: a candidate gene based analysis of InDel polymorphisms in goats. Cytokine. (2019) 115:109–15. 10.1016/j.cyto.2018.11.024 30477986

[144] Hasenauer FC Rossi UA Caffaro ME Raschia MA Maurizio E Poli MA. Association of TNF rs668920841 and INRA111 polymorphisms with caprine brucellosis: a case-control study of candidate genes involved in innate immunity. Genomics. (2020) 112:3925–32. 10.1016/j.ygeno.2020.06.050 32629097

[145] Chen Y Xu W Chen C Blair H Gao J. Variations in MHC-DRB1 exon2 and associations with Brucellosis susceptibility in Chinese Merino sheep. BioRxiv. (2016) 1–27. 10.1101/038539

